# HIV-1 subtype A infection in a community of intravenous drug users in Pakistan

**DOI:** 10.1186/1471-2334-6-164

**Published:** 2006-11-14

**Authors:** Saeed Khan, Mohammad A Rai, Mohammad R Khanani, Muhammad N Khan, Syed H Ali

**Affiliations:** 1 Department of Biological and Biomedical Sciences, Aga Khan University, Karachi, Pakistan; 2 Dow University of Health Sciences, Karachi, Pakistan

## Abstract

**Background:**

Data on the subtypes of HIV in a population help in predicting the potential foci of epidemic, tracking the routes of infection and following the patterns of the virus' genetic divergence. Globally, the most prevalent HIV infection is the HIV-1 subtype C. In Asia, predominant subtypes of HIV-1 are B, C, and CRF-01AE. During the last few years, HIV prevalence in Pakistan has taken the form of a concentrated epidemic in at least two high risk groups, namely, Intravenous Drug Users (IDUs) and Male Sex Workers (MSWs). Factors that have facilitated the proliferation of HIV infection include transmission through a large number of repatriates and needle-sharing intravenous drug users, unscreened blood transfusions, and sexual illiteracy. The HIV subtypes infecting Pakistani populations have not been explored to date. In this study, we analyzed HIV-1 subtypes from in a high-risk community of IDUs in Karachi, the largest city of Pakistan.

**Methods:**

Samples were collected from 34 IDUs after their informed consent. In addition, the study subjects were administered a questionnaire regarding their sexual behavior and travel history. For HIV analysis, DNA was extracted from the samples and analyzed for HIV types and subtypes using subtype-specific primers in a nested polymerase chain reaction (PCR). The results from this PCR were further confirmed using the Heteroduplex Mobility Assay (HMA).

**Results:**

We found HIV-1 subtype A in all the 34 samples analyzed. A few of the study subjects were found to have a history of travel and stay in the United Arab Emirates. The same subjects also admitted to having contact with commercial sex workers during their stay abroad.

**Conclusion:**

Our study therefore shows clade A HIV-1 to be prevalent among the IDUs in Karachi. As the prevalence of HIV in Pakistan continues to rise, more work needs to be done to track the infection, and to analyze the strains of HIV spreading through the country.

## Background

HIV-1, the globally prevalent type of HIV, is characterized by high genetic variability and is classified into three groups, M, O, and N. Group M strains show the highest worldwide distribution and currently comprise 11 subtypes (A1, A2, B, C, D, F1, F2, G, H, J and K) and almost 30 Circulating Recombinant Forms (CRFs). HIV subtypes that get introduced in a community tend to spread and evolve within that population. Consequently, HIV subtypes show distinct geographic distribution on regional and global levels. Knowledge of HIV-1 subtypes can therefore be used to understand the foci of HIV infection, routes of its spread, as well as the patterns of the virus' genetic divergence [1-3].

Globally speaking, Subtype A is the principal HIV-1 subtype found in Central and North African countries; Subtype B is predominant in USA, Europe, Australia, Thailand and Brazil; Subtype C is prevalent in South Africa, Ethiopia and India; CRF01_AE is common in Southeast Asia [4]. Among the Asian countries, although information on HIV subtypes is limited, distribution of HIV subtypes appears to remarkably diverse. In Iran, HIV-1 subtypes A and B have been reported among, respectively, IDUs and hemophiliacs [5]. In Uzbekistan, IDUs have been found infected with HIV-1 subtype A as well as 03_CRFAB [6], whereas other cases of CRF02_AG have also been reported [7]. In Kazakastan, as well, IDUs were reported to have HIV-1 subtype A [8]. Among the Islamic countries, Yemen has HIV-1 subtypes A, B, C, and D, as well as some unique recombinant forms (URFs) [9], Turkey has reported subtypes A, B, C, D, as well as F [10]. In Lebanon, subtypes of both HIV-1 (subtypes A, B, C, D, and G) and HIV-2 (subtype B) have been reported [11].

In Pakistan, no HIV subtyping data is available so far. In terms of HIV infection, Pakistan is a high-risk, low-prevalence country, now with pockets of concentrated epidemics in certain high risk groups. HIV high-risk populations in Pakistan include intra- and inter-continental migrants, commercial sex workers (including male sex workers), people involved in unprotected intercourse, and individuals exposed to contaminated needles through drug users and/or at informal health-care facilities [12-15]. Among these high-risk groups, the highest prevalence, respectively, 26% and 7%, has been recorded among IDUs and MSWs (personal communication).

The study presented here is the first attempt to investigate the HIV-1 subtypes prevalent in Pakistan. For this study, we analyzed a community of IDUs residing in central Karachi, Pakistan, and representing similar racial and cultural background. According to certain estimates, in the year 2002, there were 500,000 heroin users in Pakistan, of which 60,000 were thought to be injectors [16]. As needle-sharing in such groups of IDUs is a common practice, it is logical to assume that they have infected each other with HIV. Furthermore, it may be rationalized that the HIV subtypes circulating among these IDUs derive from the same ancestral strain(s). To investigate the modes of HIV transmission in this community we administered a questionnaire among the subjects, investigating their sexual and travel history. To analyze the HIV subtypes, PCR analysis was performed, using subtype-specific primers on DNA extracted from blood. The subtyping results were further confirmed by using Heteroduplex Mobility Assay (HMA).

## Methods

### a) Collection of samples and subject history

The study was approved by the Internal Review Board of Aga Khan University, Karachi. HIV-1 positive patients were selected from a community of IDUs. 2–3 ml of whole blood was collected from these subjects with their prior oral/verbal informed consent. For all of the subjects studied, HIV-1 status was previously known to be positive, based on their HIV antibody tests. The subjects were also asked to volunteer information regarding behavior considered high risk for HIV infection. Additionally, the subjects were interviewed about their travel history outside of Pakistan.

### b) Extraction of DNA (cellular and viral)

To 0.5 ml of whole blood, 0.9 ml of 1X RBC lysing solution (0.32 M Sucrose, 1 % Triton X-100, 5 mM MgCl_2_.6 H_2_O, 12 mM Tris- HCl, pH 7.6) was added and centrifuged at 13000 rpm for 1 min. The supernatant was discarded and the pellet was re-extracted with 0.9 ml RBC lysing solution. After centrifugation at 13,000 rpm, the pellet was washed with 1 ml water and allowed to dry. To the dried pellet, 20 μl 20 % SDS, 80 μl Proteinase K buffer (0.375 M NaCl, 0.12 M EDTA, pH 8.0) and 40 μl of 10 mg/ml Proteinase K was added to the solution was incubated at 55°C for 1 hour. Subsequently, 100 μl of 6 M NaCl was added to the sample and centrifugation was carried out for 5 min at 13000 rpm. Supernatant was then transferred to a fresh tube and added with 1 ml 100% ethanol. DNA was then pellet by centrifugation at 13000 rpm for 5 min. The DNA pellet was washed with 70% ethanol, air-dried, re-suspended in 50 μl of 1X TE buffer, and stored at -20°C.

### c) PCR analysis

#### *β-globin *PCR

As a control for DNA extraction, β-globin PCR was done with PC03 and PC04 primers (Table 1). The final 25 μl PCR mixture contained 5 μl of sample, 1X PCR buffer (10 mM Tris-HCl, pH9.0, 50 mM KCl, 0.1 % Triton X-100), 1 mM MgCl_2_, 200 μM of dNTPs, 0.2 pmol of each primer, and 0.2 U of *Taq Polymerase*. Thermocycling conditions were 94°C for 5 min; 40 cycles of 94°C for 30 sec, 51°C for 30 sec, 72°C for 30 sec; 72°C for 5 min.

**Table 1:** List of primers employed in the study.

**Primer**	**Sequence**
PC03	ACACAACTGTGTTCACTAGC
PC04	CAACTTCATCCACGTTCACC
JA9AE	CACAGTACAATGCACACATG
JA9B	CACAGTACAATGTACACATG
JA12A	GCAATAGAAAAATTCTCCTC
JA12B	ACAGTAGAAAAATTCCCCTC
JA10UB	CTGTTAAATGGCAGTCTAGC
JA10UC	CTGTTAAATGGTAGTCTAGC
JA10UG	CTGTTAAATGGCAGTTTAGC
JA11LAE	AATTTCTAGATCCCCTCCTG
JA11LB	AATTTCTGGGTCCCCTCCTG
JA11LC	AATTTCTAGGTCCCCTCCTG
JA11QA	CCCCTCCTGAGGAGTTAGCA
JA11VB	CACAATTAAAACTGTGCATTACAA
JA11XC	TTGTTTTATTAGGGAAGTGTTC
JA11YE	AAATTCCCCTCTACAATTAAAATGA
	
Primers used in *gag *HMA	
HIG777	TCACCTAGAACTTTGAATGCATGGG
H1P0202	CTAATACTGTATCATCTGCTCCTGT
H1Gag1584	AAAGATGGATAATCCTGGG
g17	TCCACATTTCCAACAGCCCTTTT

PC03/PC04 primers for β-Globin, JA9AE, JA9B are the upstream primers and JA12A, JA12B are the downstream primer for the first round PCR. JA10UB, JA10UC and JA10UG are the inner upstream primers; JA11LAE, JA11LB and JA11LC are the inner downstream general primers. JA11QA, JA11VB, JA11XC and JA11YE are the inner downstream subtype specific primers for subtypes A, B, C and CRF01-AE, respectively.

Primers for *gag *HMA are relative to HIV-1 group M strain ELI (GenBank accession number K03454)

#### Nested PCR for HIV-1 subtyping

PCR was performed to confirm HIV-1 infection and to analyze the subtypes. PCR was performed as described by Kato, *et al*. [4], to distinguish subtypes A, B, C, and CRF01-AE in the *env *C2V3C3 region.

Cell-associated *env *V3 viral DNA sequences were amplified by nested PCR as follows: The reaction mixture of 25 μl for both first and second round PCR contained 10 mM Tris-HCl, pH9.0, 50 mM KCl, 0.1 % Triton X-100, 1.5 mM MgCl_2_, 200 μM dNTPs, and 0.25 U of *Taq Polymerase*. The first round PCR was performed with 0.5 μM of primers JA9AE, JA9B, JA12A and JA12B (Table 1). Thrermocycling conditions were 94°C for 5 min; 30 cycles of 94°C for 15 sec, 56°C for 30 sec, 72°C for 60 sec; 72°C for 5 min.

1 μl of the first-round PCR product was used in each second-round PCR. Upstream primers used were a mixture of 0.33 μM each of JA10UB, JA10UC, and JA10UG (Table 1). Downstream primers used were different for each reaction. For subtype-independent amplification, a mixture of three primers was used, namely, 0.33 μM each of JA11LAE, JA11LB, and JA11LC. For subtype A 1 μM JA11QA; for subtype B, 1 μM JA11VB; for subtype C, 1 μM JA11XC, and for CRF01-AE, 1 μM JA11YE. Thermocycling conditions for the second round was 94°C for 5 min; 30 cycles of 94°C for 15 sec, 60°C for 30 sec, 72°C for 60 sec; 72°C for 5 min. The amplified products were detected on a 2% agarose gel and visualized by ethidium bromide staining.

A particular subtype was assigned to a sample if that subtype-specific reaction produced a band higher than 20% in intensity of the band produced by the subtype-independent reaction [4].

#### Heteroduplex Mobility Assay (HMA)

To confirm the results obtained from PCR we used Heteroduplex Mobility Assay (HMA)[17]. The assay was performed using a kit from the NIH AIDS Research and Reference Reagent Program (Rockville, MD, USA). The HMA was performed for the HIV-1 *gag *region, according to the protocol provided with the kit. For some selected samples, we further confirmed our results thorough another HMA for the *env *region. These confirmatory HMAs were independently performed at James I. Mullins' laboratory at the University of Washington School of Medicine, Seattle, USA. For *gag *HMA, nested PCR was used to amplify 1.121 kb and then 0.448 kb internal fragment of the HIV-1 Gag encoding region from both the uncharacterized sample and known reference strain. Primers HIG777 and H1P0202 were used in the first round while H1Gag1584 and g17 were used in the second round PCR (Table 1).

Upon completion of PCR, 4.5 μl the gag reference amplicon was mixed with 4.5 μl of gag amplicon of the unknown sample, along with 1 μl of 10X Heteroduplex annealing buffer (1 M NaCl, 20 mM EDTA,100 mM Tris.HCl pH7.8). The mixture was heated at 95°C for 2 minutes, followed by rapid cooling on ice. 3 μl loading dye was added to the mix and ran on 5% polyacrylamide containing 20% urea, for 3–4 hours at 250 V. Subsequently, the gel was stained with ethidium bromide and DNA bands visualized by UV illumination.

## Results

**Figure 1:**
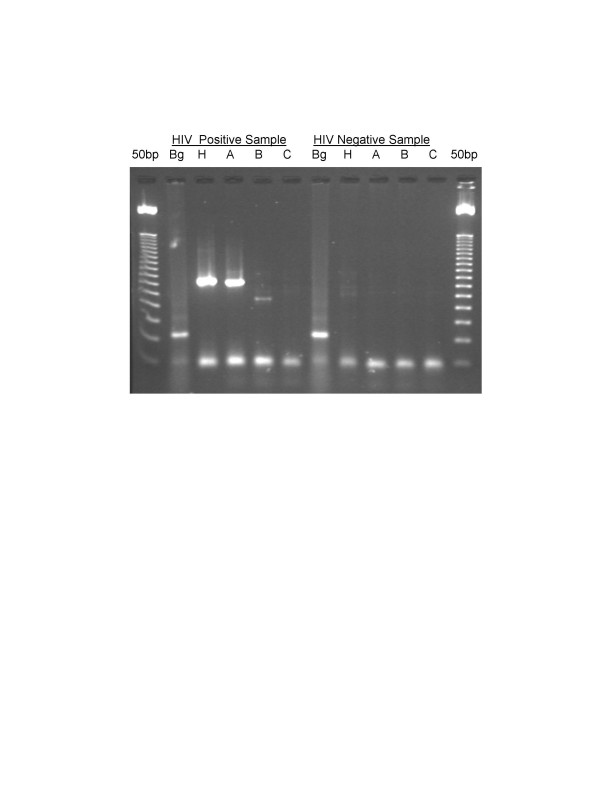
Representative gel of subtype-specific PCR for HIV-1. Symbols indicate the primers used: Bg; β-globin. H; subtype-independent PCR for HIV-1; A, B, C; respectively, HIV-1 subypes A, B and C. The first and last lanes represent 50 bp molecular weight ladder. Two panels are shown, i.e., PCR amplification from known HIV positive and HIV negative subjects. In the HIV negative subject, only the β-globin gene was amplified. In the HIV positive sample, products were observed for subtype-independent (H), and HIV-1 subtype A specific PCR. A faint band is observed in lane B, but, as explained in *Material and Methods*, this band was disregarded due to its significantly low intensity as compared to the band in H (and A).

**Figure 2:**
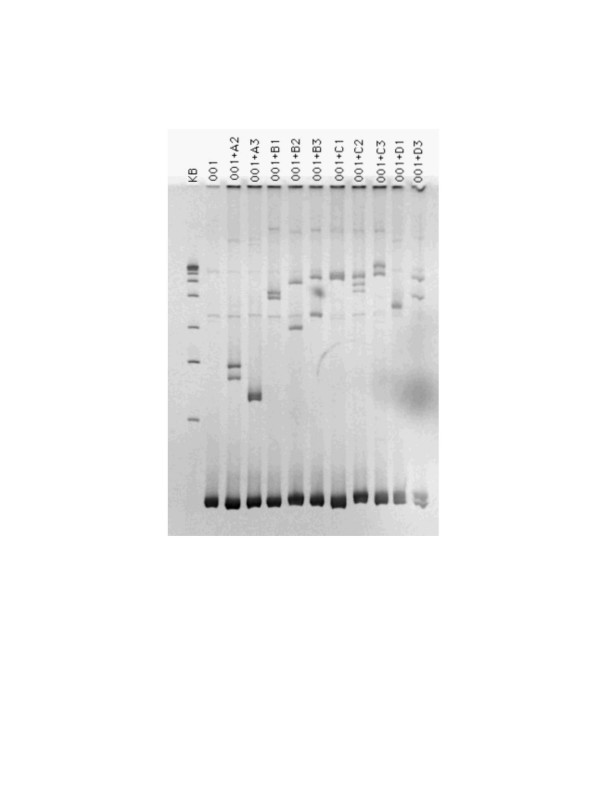
Representative *gag *HMA gel. A2, A3, B1, B3, C1, C3, D1, D3, represent reference HIV-1 subtypes whose *gag *gene was partially amplified, and mixed/melted/annealed with the unknown sample (001) to form heteroduplexes. KB and 001 represent, respectively, the molecular weight ladder and the sample alone. The gel shows the fastest migrating band in lane A3, identifying the unknown subtype to be closest related to the reference A3.

We have analyzed subtypes of HIV-1 in a group of HIV-1 positive IDUs using two different techniques, i.e., subtype-specific PCR (Fig. 1) and HMA (Fig. 2). Blood samples from 34 HIV positive subjects were analyzed for this study and they were all found to have HIV-1 subtype A (Figs. 1&2). Some of the IDU subjects had a history of travel and prolonged stay abroad, and of sexual contact with commercial sex workers during their stay abroad (Table 2).

**Table 2:** History of the subjects studied.

IDUs	34
Prolonged stay abroad	3
Contact with CSW	2*
Travel History:	
Dubai	1*
Sharja	1*
Iran	1

Out of the three IDUs who had a history of prolonged (more than one year) stay abroad, two (marked by asterisks) admitted to sexual contact with commercial sex workers (CSW) during their stay abroad.

## Discussion

The work presented here is the first report on the prevalent subtypes of HIV in Pakistan. Since the official recognition of the first case of an HIV infected individual in 1986 in Pakistan [18], the number of persons infected with HIV and/or AIDS has been constantly on the rise. Being an underdeveloped country with low (sexual) literacy rate, Pakistan is thought to have an overwhelming number of risk factors, including gender bias, poverty, substandard healthcare, and prevailing social taboos on sexual awareness (16) that may contribute to the spread of HIV in the country. Thus, due to the low number of reported cases but a high degree of risk factors, Pakistan is considered a low prevalence yet high-risk country. There have been reported outbreaks of viral infections, including HIV [19,20], in various pockets of Pakistan. No attempts have yet been made, however, to track the origin of infection, or to monitor its spread.

One way of tracking a viral infection is by establishing the epidemiology of its subtypes. This kind of study, however, has not so far been undertaken in Pakistan. Consequently, it is all a matter of speculation when it comes to identifying the sources of infection outbreaks. Understandably, therefore, well-intentioned attempts at screening and/or prevention of HIV are rendered ineffective due to a lack of pin-pointed focus.

Demography of HIV high risk groups in Pakistan is typical of many other countries, with the highest prevalence of HIV found among sex workers, needle-sharing drug addicts, and recipients of unscreened blood products [21]. In the last few years, however, HIV prevalence has gradually risen among the IDUs [19,22-28]. Our communication with the HIV Surveillance Centers in the country reveals that the prevalence of HIV infection in the community of IDUs (mainly located in the central city of Karachi) has risen to 26%. This upgrades the occurrence of HIV in IDUs from low prevalence to a concentrated epidemic.

Work on the analysis of HIV-1 subtypes in Pakistan must continue. As data on HIV subtypes in the neighboring Asian countries [5-8], and Islamic countries in Europe [10] and Middle East [9,11] start to accumulate, patterns of viral spread and divergence are beginning to emerge. In the long run, subtype data on HIV will be tremendously useful for determining the right treatment and prevention strategies as well as in designing vaccine trials, when an HIV vaccine becomes available.

## Conclusion

With the work reported here, we have initiated fundamental groundwork that shall lead to more educated attempts at prevention and control of HIV infection in Pakistan. As the prevalence of HIV in Pakistan continues to rise, the need to identify, quantify, and track the virus in high-risk communities warrants urgency.

## Competing interests

The author(s) declare that they have no competing interests.

## Authors' contributions

SA carried out all the experimental work. MAR and MNK assisted with experimental work and in data interpretation. MRK helped in the collection of patient samples and in the administration of questionnaire survey. SHA supervised the work and the writing of manuscript. All authors have read and approved the final manuscript.

## Pre-publication history

The pre-publication history for this paper can be accessed here:



## References

[B1] Potter SJ, Chew CB, Steain M, Dwyer DE, Saksena NK (2004). Obstacles to successful antiretroviral treatment of HIV-1 infection: problems & perspectives. Indian J Med Res.

[B2] Coutinho RA, Prins M, Spijkerman IJ, Geskus RB, Keet RP, Fennema HS, Strathdee SA (1996). Summary of track C: epidemiology and public health. Aids.

[B3] van der Groen G, Nyambi PN, Beirnaert E, Davis D, Fransen K, Heyndrickx L, Ondoa P, Van der Auwera G, Janssens W (1998). Genetic variation of HIV type 1: relevance of interclade variation to vaccine development. AIDS Res Hum Retroviruses.

[B4] Kato S, Saito R, Hiraishi Y, Kitamura N, Matsumoto T, Hanabusa H, Kamakura M, Ikeda Y, Negishi M (2003). Differential prevalence of HIV type 1 subtype B and CRF01_AE among different sexual transmission groups in Tokyo, Japan, as revealed by subtype-specific PCR. AIDS Res Hum Retroviruses.

[B5] Sarrami-Forooshani R, Das SR, Sabahi F, Adeli A, Esmaeili R, Wahren B, Mohraz M, Haji-Abdolbaghi M, Rasoolinejad M, Jameel S, Mahboudi F (2006). Molecular analysis and phylogenetic characterization of HIV in Iran. J Med Virol.

[B6] Kurbanov F, Kondo M, Tanaka Y, Zalalieva M, Giasova G, Shima T, Jounai N, Yuldasheva N, Ruzibakiev R, Mizokami M, Imai M (2003). Human immunodeficiency virus in Uzbekistan: epidemiological and genetic analyses. AIDS Res Hum Retroviruses.

[B7] Carr JK, Nadai Y, Eyzaguirre L, Saad MD, Khakimov MM, Yakubov SK, Birx DL, Graham RR, Wolfe ND, Earhart KC, Sanchez JL (2005). Outbreak of a West African recombinant of HIV-1 in Tashkent, Uzbekistan. J Acquir Immune Defic Syndr.

[B8] Bobkov AF, Kazennova EV, Sukhanova AL, Bobkova MR, Pokrovsky VV, Zeman VV, Kovtunenko NG, Erasilova IB (2004). An HIV type 1 subtype A outbreak among injecting drug users in Kazakhstan. AIDS Res Hum Retroviruses.

[B9] Saad MD, Al-Jaufy A, Grahan RR, Nadai Y, Earhart KC, Sanchez JL, Carr JK (2005). HIV type 1 strains common in Europe, Africa, and Asia cocirculate in Yemen. AIDS Res Hum Retroviruses.

[B10] Yilmaz G, Midilli K, Turkoglu S, Bayraktaroglu Z, Kuskucu AM, Ozkan E, Atasever L, Calangu S, Altas K (2006). Genetic subtypes of human immunodeficiency virus type 1 (HIV-1) in Istanbul, Turkey. Int J Infect Dis.

[B11] Pieniazek D, Baggs J, Hu DJ, Matar GM, Abdelnoor AM, Mokhbat JE, Uwaydah M, Bizri AR, Ramos A, Janini LM, Tanuri A, Fridlund C, Schable C, Heyndrickx L, Rayfield MA, Heneine W (1998). Introduction of HIV-2 and multiple HIV-1 subtypes to Lebanon. Emerg Infect Dis.

[B12] Mujeeb SA, Altaf A (2003). The AIDS pandemic. N Engl J Med.

[B13] Altaf A, Mujeeb SA (2002). Unsafe disposal of medical waste: a threat to the community and environment. J Pak Med Assoc.

[B14] Hyder AA, Khan OA (1998). HIV/AIDS in Pakistan: the context and magnitude of an emerging threat. J Epidemiol Community Health.

[B15] Baqi S, Nabi N, Hasan SN, Khan AJ, Pasha O, Kayani N, Haque RA, Haq IU, Khurshid M, Fisher-Hoch S, Luby SP, McCormick JB (1998). HIV antibody seroprevalence and associated risk factors in sex workers, drug users, and prisoners in Sindh, Pakistan. J Acquir Immune Defic Syndr Hum Retrovirol.

[B16] Kuo I, Ul-Hasan S, Galai N, Thomas DL, Zafar T, Ahmed MA, Strathdee SA (2006). High HCV seroprevalence and HIV drug use risk behaviors among injection drug users in Pakistan. Harm Reduct J.

[B17] Barlow KL, Green J, Clewley JP (2000). Viral genome characterisation by the heteroduplex mobility and heteroduplex tracking assays. Rev Med Virol.

[B18] Khanani RM, Hafeez A, Rab SM, Rasheed S (1988). Human immunodeficiency virus-associated disorders in Pakistan. AIDS Res Hum Retroviruses.

[B19] Shah SA, Altaf A, Mujeeb SA, Memon A (2004). An outbreak of HIV infection among injection drug users in a small town in Pakistan: potential for national implications. Int J STD AIDS.

[B20] Aziz S, Memon A, Tily HI, Rasheed K, Jehangir K, Quraishy MS (2002). Prevalence of HIV, hepatitis B and C amongst health workers of Civil Hospital Karachi. J Pak Med Assoc.

[B21] Khawaja ZA, Gibney L, Ahmed AJ, Vermund SH (1997). HIV/AIDS and its risk factors in Pakistan. Aids.

[B22] Zafar T, Brahmbhatt H, Imam G, ul Hassan S, Strathdee SA (2003). HIV knowledge and risk behaviors among Pakistani and Afghani drug users in Quetta, Pakistan. J Acquir Immune Defic Syndr.

[B23] Ahmed MA, Zafar T, Brahmbhatt H, Imam G, Ul Hassan S, Bareta JC, Strathdee SA (2003). HIV/AIDS risk behaviors and correlates of injection drug use among drug users in Pakistan. J Urban Health.

[B24] Strathdee SA, Zafar T, Brahmbhatt H, Baksh A, ul Hassan S (2003). Rise in needle sharing among injection drug users in Pakistan during the Afghanistan war. Drug Alcohol Depend.

[B25] Agha A, Parviz S, Younus M, Fatmi Z (2003). Socio-economic and demographic factors associated with injecting drug use among drug users in Karachi, Pakistan. J Pak Med Assoc.

[B26] Haque N, Zafar T, Brahmbhatt H, Imam G, ul Hassan S, Strathdee SA (2004). High-risk sexual behaviours among drug users in Pakistan: implications for prevention of STDs and HIV/AIDS. Int J STD AIDS.

[B27] Emmanuel F, Akhtar S, Attarad A, Kamran C (2004). HIV risk behavior and practices among heroin addicts in Lahore, Pakistan. Southeast Asian J Trop Med Public Health.

[B28] Shah SA, Altaf A (2004). Prevention and control of HIV/AIDS among injection drug users in Pakistan: a great challenge. J Pak Med Assoc.

